# Human unintentional and intentional interpersonal coordination in interaction with a humanoid robot

**DOI:** 10.1371/journal.pone.0261174

**Published:** 2022-01-19

**Authors:** Ghiles Mostafaoui, R. C. Schmidt, Syed Khursheed Hasnain, Robin Salesse, Ludovic Marin

**Affiliations:** 1 Neurocybernetic team, ETIS ENSEA, CNRS, CY University, Cergy-Pontoise, France; 2 Department of Psychology, College of the Holy Cross, Worcester, Massachusetts, United States of America; 3 EuroMov Digital Health in Motion, Montpellier University, IMT Mines Ales, Montpellier, France; Georgia State University, UNITED STATES

## Abstract

In order to establish natural social synchrony between two humans, two requirements need to be fulfilled. First, the coupling must be bi-directional. The two humans react to each other’s actions. Second, natural social bodily synchronization has to be intentional or unintentional. Assuming that these essential aspects of human-human interactions are present, the present paper investigates whether similar bodily synchrony emerges between an interacting human and an artificial agent such as a robot. More precisely, we investigate whether the same human unintentional rhythmic entrainment and synchronization is present in Human Robot Interaction (HRI). We also evaluate which model (e.g., an adaptive vs non adaptive robot) better reproduces such unintentional entrainment. And finally, we compare interagent coordination stability of the HRI under 1) unidirectional (robot with fixed frequency) versus bidirectional (robot with adaptive frequency) rhythmic entrainment and 2) human intentional versus unintentional coupling. Fifteen young adults made vertical arm movements in front of the NAO robot under five different conditions of intentional/unintentional and unidirectional/bidirectional interactions. Consistent with prior research investigating human-human interpersonal coordination, when humans interact with our robot, (i) unintentional entrainment was present, (ii) bi-directional coupling produced more stable in-phase un-intentional and intentional coordination, (iii) and intentional coordination was more stable than unintentional coordination. To conclude, this study provides a foundation for modeling future social robots involving unintentional and bidirectional synchronization—aspects which seem to enhance humans’ willingness to interact with robots.

## Introduction

Social interaction is a hallmark of being human. Although people generally interact through language, it is now well known that they also coordinate their bodies during interactions [1]. For instance, individuals can be synchronized with someone when speaking with a partner [2] or match someone’s expressions when the person front of us yawns (e.g. [3]. As long as there is a perceptual contact between the two partners during an interaction, bodily coordination is very often present in our everyday life (e.g., Issartel et al., 2007). It is even difficult to not fall into the bodily coordination with someone moving in front of us [4]. Sometimes such interpersonal coordination is intentional. In this case the two (or more) individuals are voluntarily moving in synchrony together. For instance, when soldiers are marching together, they all intend to be perfectly coordinated. Even if one of them is off rhythm for one cycle, he/she immediately changes his/her step to be back in synchrony. The same phenomenon occurs when two people shake their hands during greetings, or even in a more complex sport settings when performing routines on a trampoline. Research has found that the dynamics underlying synchronized interpersonal coordination are the same as those governing the synchronization of physical oscillators. Schmidt and colleagues in the 1990s found that interpersonal rhythmic coordination, like bimanual interlimb coordination has two main coordination modes: in-phase (two limbs move in the same direction) or in antiphase (the two limbs move in the opposite direction). The in-phase mode is more stable than the antiphase pattern [5] and an abrupt transition occurs when the frequency increases from antiphase to in phase [6]. Coupled oscillator dynamics were used to model these two stationary modes [7] as well as the antiphase breakdown [8]. A more common type of interpersonal coordination than intentional coordination occurs in our everyday life, namely, unintentional (spontaneous or involuntary) coordination: When people interact, their bodily movements tend to be spontaneously entrained. Such synchrony can be observed, for example, when two people having a conversation are walking in the street in synchrony even if they both have different preferred stride frequency. Yet, as soon as these two persons separate in different direction, they immediately walk back to their own preferred frequency. Schmidt and O’Brien (1997) were the first to formalize this phenomenon in an experiment in which two participants sitting next to each other were instructed to swing a handheld pendulum at their own frequency either while watching or not watching (control condition) the other person’s pendulum [9]. The results showed that participants became synchronized with each other when they were watching each other’s pendulum even though they did not intend to: They became spontaneously coordinated without being aware of it. In a follow-up experiment, Issartel et al. (2007) asked participants explicitly to NOT synchronize with a participant sitting front of them while improvising forearm movements. The results demonstrated that even if participants reported the other individual did not influence them, they could not avoid synchronizing with their partner. The conclusion is that people become spontaneously and unintentionally coordinated when someone with whom they are interacting is moving in front of them [4]. Intentional and unintentional interpersonal coordination share some common properties. First there is no interpersonal coordination without any information exchange between the two systems [10]. In intrapersonal coordination, this coupling can be neural or mechanical, but in the case of interpersonal interaction, this exchange is perceptually based. This perceptual exchange can occur through visual [11], auditory [12] or tactile information [13]. For instance, in terms of visual information, several articles have shown that some visual cues are better suited to establish interpersonal coordination. Participants exhibited greater unintentional coordination and more stable intentional coordination with a stimulus when they tracked it with their eyes [11]. When a moving partner is in peripheral view, the unintentional coordination is weaker than when in central view [14]. Finally, Hajnal et al. (2012) found that the amount and location of available visual information influence the stability of the observed synchronization [15]. The second common property that intentional and unintentional interpersonal coordination share is that coordination performance is stronger in a bidirectional coupling than in unidirectional one. Bidirectional coupling occurs when the two participants are influenced by each other. When one is acting, the other one is reacting based on the action of the first one and so on [16]. For instance, this phenomenon occurs when two persons are carrying a table together. They both have to take into account the movements of the other one in order to fulfill the task [17]. A bidirectional coupling facilitates the maintenance of a stable phase relationship [18] in performing music. In contrast a unidirectional coupling entails the adaptation of only one of the participants. For instance, if rider B follows rider A but the A does not follow B. B is the only one who adapts. Not surprising a unidirectional coupling leads to a weaker coordination because one participant supports the synchronization task. Several studies have experimentally demonstrated such a phenomenon. Demos et al. (2017) showed that in music, bidirectional coupling yields optimal coordination between musicians, compared with unidirectional coupling as would arise when a performer plays with a recording. Both (bidirectional and unidirectional coordination) have been dynamically modeled to study their different coordination phenomena [19]. According to the dynamical system approach, bidirectional coordination is the optimal state during live performance. The HKB model (named after the initials of the authors; Haken, Kelso and Bunz, 1985) also formalized this coupling [8]. Two oscillators are both coupled such that they influence each other. However, some studies have also dynamically modeled the unidirectional coordination (between a limb and a non-adaptive environment) where the visual information of the rhythmic stimulus is considered as the energy injected into an oscillatory system and where the movements of the participants adapt to the conditions of the oscillating environmental stimulus [20]. Although intentional and unintentional interpersonal coordination share common properties, there are significant differences between these two modes. The most important and obvious is that unintentional coordination is less stable than intentional mode. Intentional coordination is promoted by the willingness of both participants to act together. If both are motivated, they can coordinate almost perfectly together, in what is called absolute coordination [21]—at same tempo with a steady phase relation between them. However, in a social interaction where bodily coordination is not the intended goal, one observes entrainment and hence coordination but without a steady phase relation because the movements are often at different tempos [22]. This weaker form of coordination is called relative coordination [21]. What ones sees (e.g., in Schmidt and O’Brien study, 1997) is metastable entrainment around the stable relative phase attractors of 0° and 180° specific to the in-phase and antiphase modes respectively. Note though that, if the two participants move in very different frequencies (for instance one with high frequency and the other one with low frequency), it is possible that sometimes there is no synchronization at all because the frequency difference is too important for the coupling strength to counterbalance such a discrepancy [22]. The best range for observing unintentional coordination is when the two subjects move within +-10% of their preferred frequency [10]. For frequency differences greater than this, the coordination takes longer to arise or might not even occur at all.

All the studies previously reported depicted inter-humans’ coordination. As artificial agents have been more and more studied in recent years, social robots have become more and more familiar, consequently, one can ask what happens if a person interacts with artificial agents such as robots. Are humans able to similarly synchronize with robots in general? Does this Human-Artificial Agent dyad follow the same general rules as human-human interpersonal coordination? Can Unintentional rhythmic entrainment occur when interacting with a robot? Do human and robot behaviors bring more stable coordination? We propose to address these questions in the current study.

Keeping in view the importance of synchrony in social interaction, synchrony has also been widely studied and used in robotics. In fact, interpersonal coordination and synchrony have been analyzed from different points of views in the robotics domain. First in terms of imitation games, interpersonal sensorimotor dynamics have been used for studying different aspects of social interaction: Simulated agents’ motor coupling and turn-taking [23], imitation detection by building interpersonal maps [24], and learning using synchrony as an internal reward [25] or as reinforcement signal to learn new sensorimotor association [26]. For example, in Hiolle et al. (2010) authors hypothesized that a constant rhythm is an intrinsic property of a positive interaction whereas a breakdown reflects a negative event [27]. Human Subjects were asked to teach a NAO robot to mirror their actions. The results suggest that “when the subjects do behave naturally, the rhythm and its variations truly reflect how well the interaction is going”. The engagement and attention during Human Robot Interaction (HRI) have also been enhanced by using synchronized multi-modal sensory signals [28–30]. Moreover, coordinated human-robot physical interactions as shown, for example, in different studies on human-robot handshaking [31, 32] or in recent experiments on robotic systems for human movement rehabilitation [33, 34] have used the idea of synchrony. Finally, and in the same line concerning assistive robots for health care, several authors argue that synchrony and reciprocity are key mechanisms which affect both behavioral (movements) and social level (relationships; [35]. For example, recent experiments with patients suffering of interactional deficit (schizophrenia) were able to coordinate with the iCub robot as well as non-patient participant [36].

Nonetheless, despite the overwhelming importance of synchrony and interpersonal coordination highlighted by the above numerous and different studies, questions on its effectiveness, significance and characteristics in the case of HRIs (comparing to human social interactions) are still under debate. Even totally opposite claims can be observed in human-robot interaction studies. In fact, Kilner et al. (2003) has suggested that robots do not trigger any interference (suggesting that interference is a sort of coordination) when interacting with a human [37]. However, the authors used in this case a non-humanoid arm robot. Oztop et al. (2005) reproduced the same kind of experiment with a real humanoid robot. In such a context, they showed humans were as much influenced by the robot as when facing humans [38]. Similarly, Shen et al. (2011) studied motor inference and motor coordination in human-humanoid interactions for different types of visual stimuli (robot, pendulum and moving dot). The authors concluded that participants tended to synchronize with agents having a human-like appearance, which means that a robot perceived as close as possible to a social entity may facilitate Human-Robot Interaction (HRI) [39]. On the other hand, other studies have argued that motor resemblance and motor resonance are more important than physical appearance. Marin et al. (2009) underlined that motor resonance between robots (humanoid) and humans could optimize the social competence of HRIs [16]. Other findings indicate that perceptions of the lifelikeness can be manipulated by simple changes, such as variation in the repertoire of motions, coordination or robot’s synchrony [40]. Lehmann et al. (2015) used a non-anthropomorphic robot with a limited range of expressive motions. They found that positively synchronized movements during an object-oriented task were interpreted by participants as engagement and created a positive disposition towards the robot. The authors conclude that “synchronization of body movements can be a powerful means to enhance the positive attitude towards a non-anthropomorphic robot” [41].

Although there are myriad of articles on human-robot coordination, most of these studies focused on intentional coordination. In order to build robots that can interact naturally with humans to be even more accepted companions, we contend that there is a need to better study coordination in HRI by comparing it with human social coordination characteristics. Especially, given the fact that unintentional coordination is so important in our social interactions (e.g., Issartel et al., 2007; Schmidt & Richardson, 2008), we argue that human unintentional coordination with a robot must be taken into account. To our knowledge, no studies have ever analyzed unintentional coordination between a human and a robot.

We hypothesis that, as for human-human interaction, human unintentional coordination is present and of importance in HRI. The present study aims at confirming such an assumption by analyzing, during human robot interactions, human intentional and unintentional coordination in both unilateral interaction (robot moving with a fixed rhythm) and bi-directional interaction (the robot is adapting to the human rhythm). In addition to the above state of the art, recent seminal works and results motivate our subject. In fact, several articles have shown that humans intentionally and unintentionally synchronize with a non-social sensory signal such as a perceived object moving on a screen (e.g., [11]. However not only did they not evaluate humanoid robots in an unintentional context but they also did not used adaptative sensory signal to allow a bidirectional interaction. In these articles, the same oscillatory dynamical patterns are observed when interacting with a human or a non-social stimulus (e.g., a moving dot). Moreover, the same dynamical coupled oscillatory equations have been used to model those patterns. For instance, two preferred modes of in-phase and antiphase [11] and an abrupt transition when increasing the frequency of the limb [42] for intentional coordination tasks. In addition, one observes less stable synchrony for unintentional tasks and a degree of metastability that depends on the tempo difference between the two movements [11]. This latter outcome verifies that there is only a small range of frequency differences for which unintentional coordination emerges with a rhythmical environment [43]. Although these studies did not analyze human intentional and unintentional coordination while interacting with robots, they clearly demonstrated the presence of the phenomenon when the sensory signal is not produced by humans, which emphasizes the fact that the same result must be obtained when a person is facing a robot. Moreover, bi-directional interactions were not considered in these studies since the moving dots or objects never adopted the subject’s rhythms.

Besides, in our recent works [44, 45] we observed evidence of human unintentional rhythmic entrainment during HRI using a robot able to synchronize its behavior to the human subject one (bi-directional interaction). Even if we consider these studies as sort of proof of concept, we didn’t analyze the phenomena in a clear way to really confirm or deny its presence as no effective statistical analysis have been conducted. Besides, no comparison has been made between intentional and unintentional behaviors.

Consequently, the objective of the present study is to more precisely analyze human interpersonal coordination while interacting with a robot. We specifically address the following questions: i) Can we confirm observation of human unintentional rhythmic entrainment and synchronization in HRI? ii) if so, which robot behavior (e.g., adaptive vs non adaptive robot) best promote such a phenomenon? And finally, iii) How to compare HRI with human-human interactions in terms of interpersonal coordination stability regarding two different aspects: 1) unidirectional (robot with fixed frequency) versus bidirectional (robot with adaptive frequency) rhythmic entrainment and 2) human intentional versus unintentional behavior?

## Method

### Participant

Fifteen young adults (8 men, 7 women) from Montpellier University participated in the experiment. Their ages were between 18 and 25 and their heights were between 1.56 m to 1.9 m. Fourteen of the participants were right-handed and one was left-handed. All the subjects were healthy with no reported physical, psychological or neurophysiological impairments.

The present study is a part of a Global Research Project on Human interpersonal coordination in interaction with a Humanoid Robot including Healthy People and Schizophrenia Patients. All participants provided written informed consent, prior to the experiment approved by the National Ethics Committee (CPP Sud-Ouest et Outre Mer—II, Toulouse, France, #2–18-65 and ID-RCB-2018-A02237–48) and conforming to the Declaration of Helsinki. In accord with identifying information policies, written informed consent for publication of identifying information/images was obtained.

### Experimental setup

**Fig 1:**
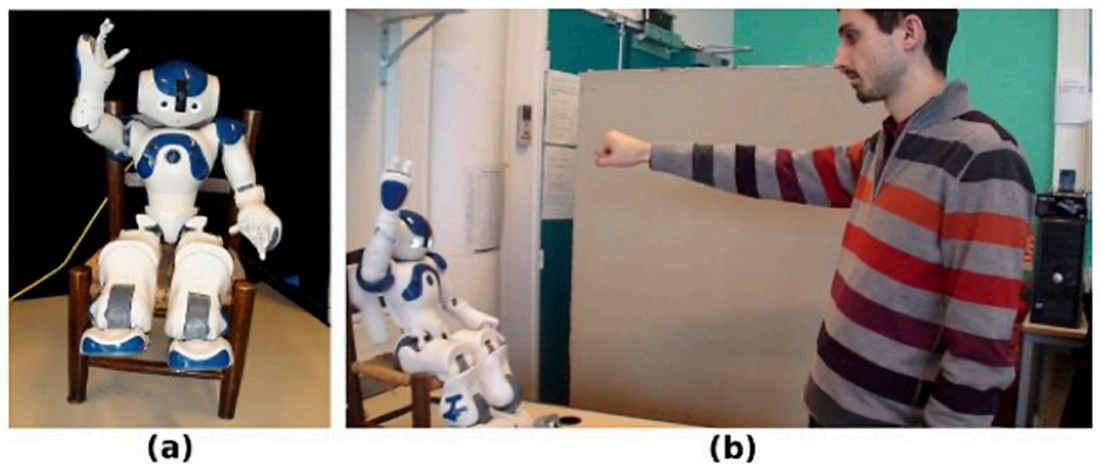
Experimental setup. (a) the NAO robot, (b) NAO Interacting with a human subject.

The experimental setup was similar to the one depicted in Fig 1 (the individual who appears in the figure has given written informed consent, as outlined in PLOS consent form, to publish these case details). The Nao robot was controlled with an external laptop running the neural model detailed below and permitting the robot to perform the task and, depending on conditions, to synchronize its behavior to the human partner. Nao’s vision was captured by an external camera to avoid the robot’s embedded camera frame rate limitations. The camera frame rate was set to 30 fps. Human participants stood 1.5 meters from the robot. The external camera was set at the same distance from the subjects at a height of 0.9 meters. NAO’s shoulder was 1.3 meters from the ground.

Two Wired Twin-Axis Goniometers (Biometrics Ltd) were used to measure movement signals (shoulder angular displacements), one on NAO’s shoulder and the other on participants’ shoulders. Each Goniometer had an accuracy of ±2° measured over a range of ±90°. An additional laptop was used to record the goniometers output signals at a frame rate of 100 Hz.

### The neural model controlling the robot

**Fig 2:**
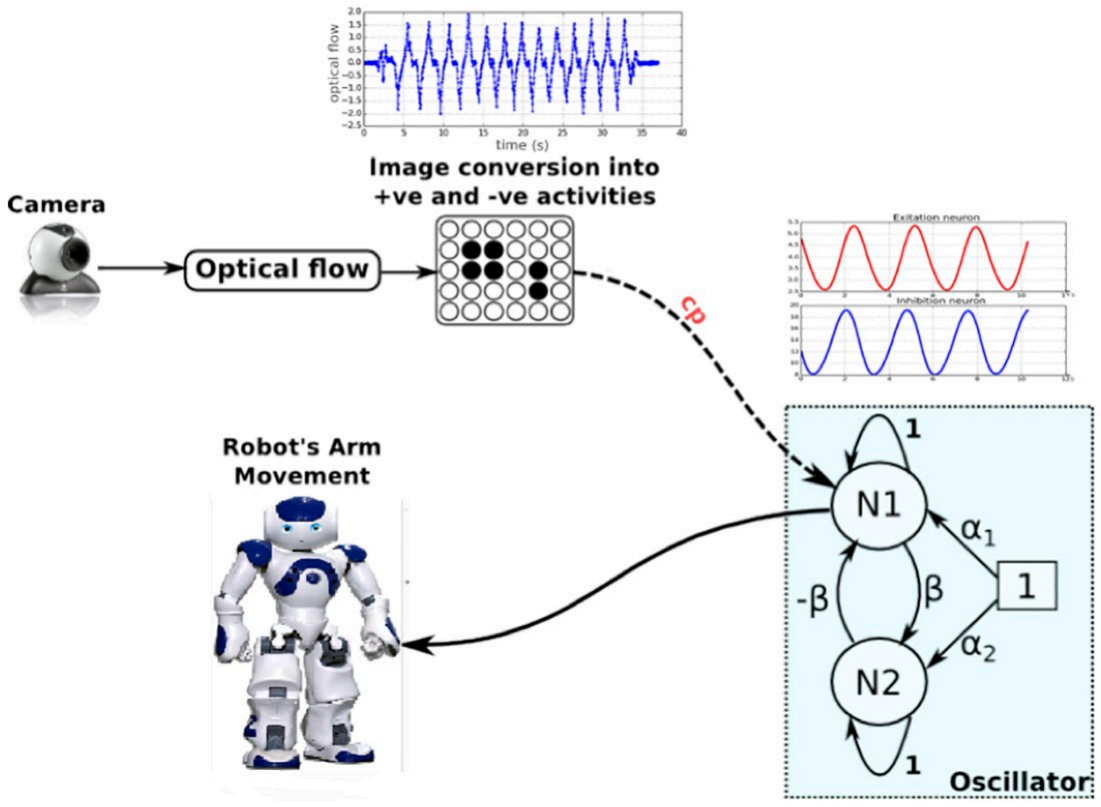
The robot’s sensorimotor controller.

As stated before, studying synchronization and rhythmic entrainment in HRI, especially in bi-directional interaction (mutual entrainment), implies first making the robot able to be rhythmically entrained by the human’s movement dynamics. Several approaches can be considered. From an ecological point of view, as introduced below, several studies have demonstrated that this interpersonal coordination is very low level and seems to be the result of a self-organizing dynamical system rather than that of a complex computational process. In dynamical systems approach, agent’s dynamics are modeled by oscillators influencing and entraining rhythmically each other [8]. Agents coordinate with each other via informational linkage using very low-level processes that can influence the mutual movement frequency and amplitude that emerges. A major difficulty in creating HRI that can produce such self-organizing dynamical system is the fact that we don’t have a direct control of both agents’ “oscillators” (human motion frequency and phase for example). To tackle this problem, in this current article, we used the entrainment effect model recently proposed by [46]. This simple architecture gave the robot the abilities to adapt its rhythms and interaction dynamics to the partner movement’s by using the optical flow induced by the human movements. This model has been validated with naive participants [44] and has been proven to be efficient to adapt, in a very simple HRI, the robot’s rhythm to the human motion dynamics (see Fig 2). It is worth noticing that other researchers have similarly investigated synchrony in HRI and other approaches can be used [28–31, 33–35, 47–51].

As depicted in Fig 2, the robot’s arm (programmed to move vertically) is controlled by a neural oscillator composed of two neurons *N*_1_ and *N*_2_ inhibiting and exciting themselves in proportion to the parameter *β* as follows:

$$\begin{array}{c} N_{1}\left(t+1\right)=N_{1}\left(t\right)-\beta N_{2}\left(t\right)+\alpha_{1} \end{array}$$ (1)

$$\begin{array}{c} N_{2}\left(t+1\right)=N_{2}\left(t\right)+\beta N_{1}\left(t\right)+\alpha_{2} \end{array}$$ (2)

where *t* represents time. To avoid the divergence of the signals, *N*2 amplitude was limited to a maximum value of 1 (if *N*_2_(*t*) > 1, *N*_2_(*t* + 1) = 1).

The oscillation frequency (the arm movement rhythm) is a function of *β*. To initiate the oscillations, *N*_1_ is fed by a constant signal *α*_1_. In our experiments, *β* was set to 0.05 (to obtain an initial fixed frequency of 0.35*Hz*), we used $\alpha_{1}=\frac{\beta }{2}$ to simplify the number of modifiable parameters. *α*_2_ is used as an offset, here *α*_2_ = 0.

Only outputs from the neuron *N*_1_ are used here as a motor command.

The overall functioning of the model illustrated in Fig 2 was as follows:

- The oscillator was connected to the Nao arm (shoulder) and oscillated at its own frequency (at 0.35*Hz*) and amplitude; Nao’s shoulder angular positions are consequently controlled and defined by the oscillator outputs.
- The movement in the visual field of Nao was estimated by a classical optical flow algorithm [52], for each pixel *I*(*x*, *y*, *t*) of the image an horizontal (*U*(*x*, *y*, *t*)) and vertical (*V*(*x*, *y*, *t*)) velocity vector was obtained. As the considered movement here is only vertically swinging the arm, only the vertical component *V*(*x*, *y*, *t*) of the optical flow vector was used. A spatial integration of *V*(*x*, *y*, *t*) over time produced a varying signal denoted *F*(*t*) = ∑_*x*_ ∑_*y*_ *V*(*x*, *y*, *t*). If the perceived movements were in an ascending direction, the amplitude of *F* was going to be positive and vice versa for the descending movements. This amplitude therefore depended on the quantity of energy induced by the perceived movement;
- When an agent interacted with Nao, the oscillator controlling the robot’s arm was then modified (amplitude, frequency and phase) by the signal *F* within certain limits weighted by a coupling factor *cp* (see Fig 2).

The mathematical equation of the oscillator could then be reformulated as follows:

$$\begin{array}{c} N_{1}\left(t+1\right)=N_{1}\left(t\right)-\beta N_{2}\left(t\right)+\alpha_{1}+F\left(t\right).cp \end{array}$$ (3)

High values of *cp* induce a greater influence of visual stimuli (*F* signal) on the robot motor controller. As a result, the robot will be able to synchronize with a wide range of frequencies. Here *cp* was set to a value of 0.4. For more details on this neural model, we invite the reader to refer to [51].

### Procedure

The NAO robot can exhibit here two different behavior modes: moving its arm vertically with a pre-defined and fixed frequency or, using the neural model described below, with an adaptive frequency making it able to synchronize with human subjects.

The task of the participants was to move their arm vertically in front of the NAO robot (see description above and Fig 1) under five different conditions. In the condition 1, subjects were blindfolded and wore headphones so that they were unable to see or hear the robot. Conditions 2 and 3 were unintentional conditions and conditions 4 and 5 were intentional conditions. For all the conditions, no a priori knowledge was given to the subject regarding the way the robot was going to move or react during the interaction. For clarity sake, the different conditions will be detailed more below after describing the experimental setup and the robot’s motor controller.

In order to prevent an impact on unintentional conditions, intentional conditions were always performed after condition 1 and the two unintentional conditions. In other words, each participant performed the first 9 trials in a random and counterbalanced order (3 trials for each of the 3 first conditions including the 2 unintentional ones) before the 6 intentional trials (3 trials for each of the 2 intentional conditions in a random and counterbalanced order). All trials lasted 30 seconds.

### Conditions

As introduced previously, the task of the participants was to move their arm in front of the robot under 5 different conditions which are going to be developed below:

#### Condition 1—No Visual / Auditory Feedback

The participants were instructed to close their eyes while listening a white noise and to move vertically their arm continuously in an oscillatory manner at their preferred frequency. In this condition, the subjects were not able neither to perceive nor to hear NAO’s movement. The reason behind this choice was to ensure that the participant moves with his/her natural frequency by removing all the external visual or acoustic perturbations. Nao was controlled by the neural model detailed previously. Its arm produced vertical oscillatory movements with an initial frequency of 0.35 Hz (set by the parameter *β*). However, the coupling factor cp (coupling strength) was set to 0.4 giving the robot an ability to adapt its movement dynamics to a large range of frequencies of interaction (approximatively from 0.2 to 0.5 Hz). Through this condition, we were able to estimate the spontaneous movement frequency of each participant. Moreover, it ensured that the robot was able to synchronize with subjects’ movements without human “help”. The chosen initial frequency (0.35 Hz) was the median of the range of reachable frequencies (approximatively from 0.2 to 0.5 Hz) deduced from our previous preliminary experiments (using the same neural model) where a large majority (but not all) of subjects produced movement dynamics within this range.

#### Condition 2—Unintentional human in unilateral coordination (UnintentionalUni)

As in the previous condition, the participants moved their arm vertically in an oscillatory manner at their preferred natural frequency. In addition, the participants were instructed to focus their visual attention on the robot’s arm but to stay at their preferred frequency (this kind of instruction was setup to trigger an unintentional coordination; see e.g., Schmidt & O’Brien 1997’s method [9]. In this condition, Nao’s camera was turned off. Consequently, the robot moved its arm with oscillatory movements corresponding to the fixed initial frequency of 0.35 Hz and hence never adapted the participant’s arm rhythm (since it could not perceive the external stimuli). In this condition, NAO’s arm could be considered as a pendulum oscillating at a fixed frequency.

#### Condition 3—Unintentional human in bi-directional coordination (UnintentionalBi)

This condition was similar to the second one except that NAO’s camera was turned on. NAO’s arm was then controlled in the same manner as the one in Condition 1 with capabilities to adapt its movements to the motion frequencies of the participants. The large range of accepted frequencies of interaction was limited by the coupling factor (which was set to 0.4 as in the Condition 1) and the robot mechanical capabilities.

#### Condition 4—Intentional human in unilateral coordination (IntentionalUni)

This condition was similar to the Condition 2 (Nao moving with a fixed frequency) but we asked the subjects to intentionally synchronize with the robot.

#### Condition 5—Intentional human in bi-directional coordination (IntentionalBi)

This condition was similar to the Condition 3 (Nao was able to adopt the human motion dynamics) but we asked the subjects to intentionally synchronize with the robot.

### Data filtering

We computed the shoulder angular displacements of the robot and participant by reducing the 2-dimensional signals of the goniometers to a 1-dimensional signal. In fact, since we only wanted to measure vertical movements, only the vertical component of the goniometers outputs was considered.

A Fast Fourier Transform (FFT) analysis performed on the data revealed that the frequency spectrum was essentially located below 1 Hz. Thus, we filtered the data with a second-order lowpass Butterworth filter with a 3 Hz cut-off frequency. This filtering process smooths the signals and can limit the accuracy of the movement capture, nevertheless, in the framework of this study a fine tuned measure of higher frequency movements is not needed as we are mostly interested in the global rhythmic behavior of the signals (periods/Frequencies and phases). Finally, linear trends have been removed from the obtained vectors. For each trial, we consequently obtained two signals (one for each goniometer) corresponding to the shoulder angular displacements (arms oscillations) of the participant and robot respectively. All data can be downloaded here.

### Data analysis

To determine the coordination between the robot and the human a number of measures were used. To evaluate the tempos and their variability that were exhibited in the interaction, the mean and standard deviations of the arms’ oscillation periods of the robot and human were calculated from the time intervals between maximum vertical heights of the arms. The degree and pattern of synchronization of the human’s and robot’s movements were measured by evaluating the relative phasing of their movement time series. A continuous relative phase time series was computed from the two movement time series using the Hilbert transform [53]. This measure provided an index of the relative timing of two rhythms. If rhythms were in the same direction at the same time, they were in-phase and had a relative phase angle of 0°. If rhythms going in the opposite direction at the same time, they were in antiphase and had a relative phase angle of 180°. The degree of synchronization was evaluated by the circular variance [54] of the relative phase time series. The circular variance measures the proportion of relative phase relationships visited by the two-time series. A circular variance of zero reflected that the time series never visited the same relative phase relationship more than once. Higher values of circular variance reflected that the two-time series visited a set of relative phase relationships continually and a value of 1 indicated perfect synchronization. The mean circular relative phase angle [54] was used to evaluate the pattern of synchronization. This measure, calculated from the relative phase time series, measured the degree of phase shift between the movements of the robot and human. Given the conventions used in the calculation, a negative relative phase angle meant that the robot was leading during the coordination. Additionally, to assess the pattern of synchronization that emerged, the distributions of relative phase angles formed between the movements of the robot and human were also evaluated. To determine these distributions, the frequency of occurrence of the absolute value of these relative phase angles for nine relative phase regions (0° to 20°, 21° to 40°, 161° to 180°) between 0° and 180° was calculated (see [9, 10] for more details). Phase-entrained coordination was indicated by a concentration of relative phase angles in the portions of the distribution near the 0° and 180°.

Within-subject analyses of variance were performed to evaluate timing and synchronization differences for the different conditions. For all statistical analyses, Greenhouse-Geisser adjustments for violations of sphericity were made as necessary. For post-hoc analyses, simple effect F-tests were used to analyze interactions and a LSD criterion was used to determine significant differences between individual means.

## Results

**Fig 3:**
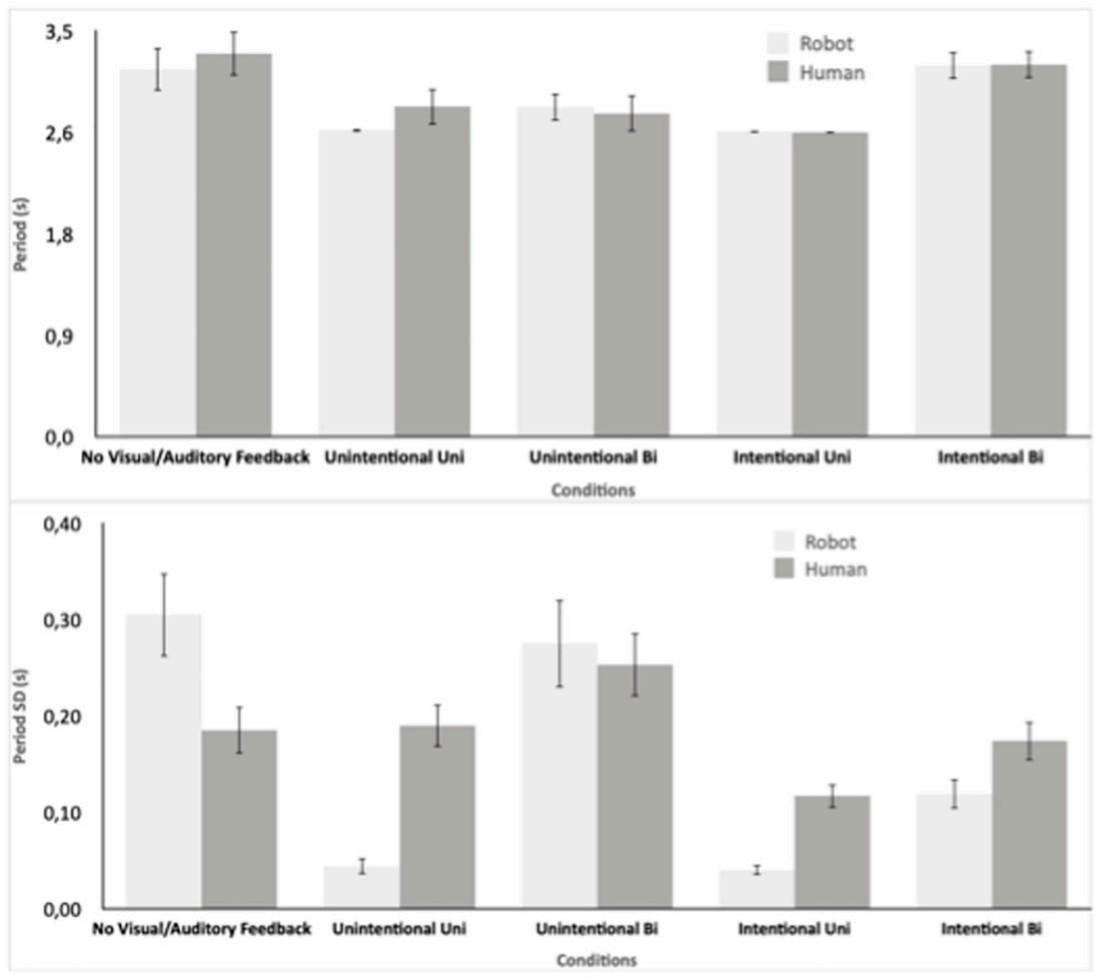
Mean and standard deviation of the arms’ oscillation periods of the robot and human.

Trials were of variable length with a mean of 66 s (*SD* = 5.27). The first 5 s of each trial were not analyzed in order to avoid any initial transient coordination. To evaluate the tempo of the coordinated movements across the five human-robot conditions, the mean period of oscillation and its standard deviation were submitted to 5 X 2 ANOVAs with within-subject variables of Condition (No Visual / Auditory Feedback, UnintentionalUni, UnintentionalBi, IntentionalUni, and IntentionalBi) and Role (Robot, Human). The analysis of mean period revealed a significant effect of Condition (*F*(2.18, 30.5) = 7.77, *p* = .001, $\eta_{p^{2}}=.36$) but no effect of Role nor a significant interaction. As seen in Fig 3, and verified by post-hoc comparisons, the No Visual / Auditory Feedback and the IntentionalBi both had significantly longer periods than the middle three conditions. In both of these conditions, the period was set by the human’s preferred period (No Visual / Auditory Feedback: *M* = 3.17*s*) which was longer than the period Nao was set to in the second and fourth conditions, UnintentionalUni and UnintentionalBi, IntentionalUni (*M* = 2.64). The period achieved in the third UnintentionalBi condition (*M* = 2.82*s*) seemed to be a compromise period for the Human and Robot preferred movement tempos.

The analysis of mean period SD revealed a significant effect of Condition (*F*(2.53, 35.4) = 17.56, *p* < .001, $\eta_{p^{2}}=.56$) and a significant interaction between Condition and Role (*F*(2.44, 34.2) = 14.74, *p* < .001, $\eta_{p^{2}}=.51$). As seen in Fig 3, and verified by post-hoc comparisons, the Robot had significantly greater variability than the human when it was directing the unilateral coupling (No Visual / Auditory Feedback) but the human had more variability when he was directing the coupling in Conditions 2 and 4 (UnintentionalUni and IntentionalUni). The human also had significantly more tempo variability in the IntentionalBi condition. However, the UnintentionalBi condition which had as much variability as the first No Visual / Auditory Feedback, showed no difference between the human and robot.

**Fig 4:**
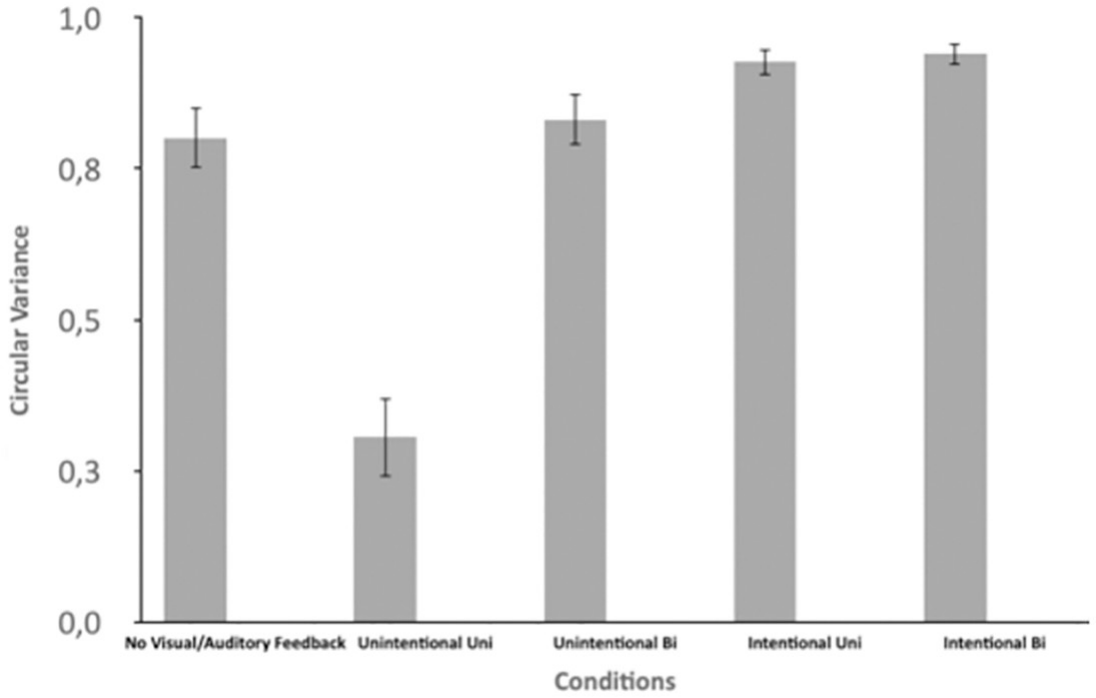
Circular variance of the relative phase time series. Note that circular variance is in unit-less and that 0 indicates no synchronization and 1 perfect synchronization.

To evaluate whether the degree of movement synchronization between the human and the robot differed across coordination conditions, a one-way ANOVA with the within-subject variable of Condition was performed. A significant effect (*F*(1.7, 23.8) = 45.6, *p* < .001, $\eta_{p^{2}}=.77$) was found (Fig 4). Post hoc comparisons revealed that the IntentionalBi had significantly higher synchronization (*M* = .94) than all other conditions while the IntentionalUni condition was second highest (*M* = .93)—significantly higher than the others. Next highest were No Visual / Auditory Feedback (*M* = .80) and UnintentionalBi (*M* = .83)—which were not different from each other but were both significantly higher than UnintentionalUni (*M* = .31). These results made sense: Intentional coordination synchronization was stronger than unintentional whereas a bi-directional coupling was stronger than a unidirectional coupling. Moreover, the unidirectional robot coupling was stronger than unintentional unidirectional human coupling because the strength of the former was set a priori (high coupling factor). Note that the unidirectional robot coupling could be changed to attain a value that was weaker than the one seen in the UnintentionalUni condition.

**Fig 5:**
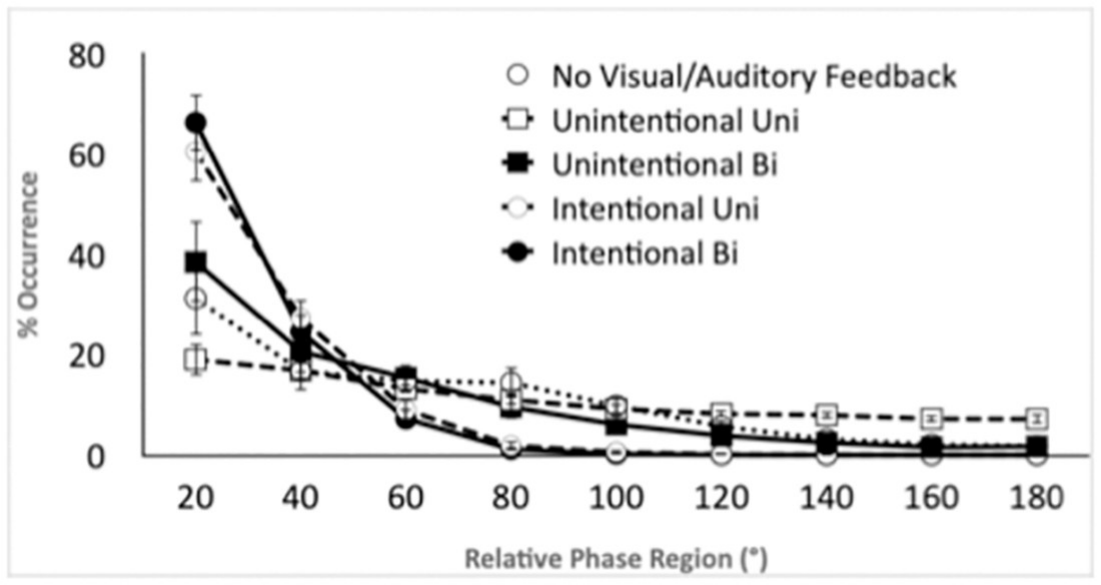
Distributions of relative phase angles of the robot and human arm’s movements.

To evaluate how the pattern of movement synchronization differed across coordination conditions, the distribution of relative phase angles across the nine phase regions was submitted to a 5 X 9 ANOVA with the within-subject factors of Condition and Phase Region. A flat distribution of relative phase angles across all phase regions would suggest no coordination occurred between the two oscillations whereas a concentration of phase angles at specific regions would indicate that phase entrainment at that phase angle had occurred. Further, a concentration of phase angles near 0° or 180° would suggest that entrainment was in the stable modes of a coupled oscillatory dynamic [9, 55]. The analysis revealed a significant effect of Phase Region, *F*(1.8, 25.6) = 68.45, *p* < .001, $\eta_{p^{2}}=.83$, and an interaction between Condition and Phase Region, *F*(3.5, 49.6) = 9.99, *p* < .001, $\eta_{p^{2}}=.42$. As can be seen in Fig 5, the entrainment observed had more in-phase modes for the intentional coordination conditions than for the unintentional conditions. Post-hoc comparisons demonstrated that both intentional coordination conditions had more in-phase behavior in the first 20° region (*Ms* = 66.4 and 60.6) than the three first conditions (all *ps* < .05) but were not different from each other. For the unintentional conditions, the UnintentionalBi (*M* = 38.5) was found to be significantly (*p* = .05) greater than the UnintentionalUni (*M* = 19.0).

## Discussion

Before discussing the different results, it is worth noticing two important aspects of this study. The first concerns the robot’s behavior. In Conditions 2 and 4, the robot played the role of a metronome dictating the interaction tempo. On the contrary, in Conditions 1, 3 and 5 the robot was acting as an adaptive agent trying to coordinate with the human partner. Besides, the high coupling factor set in the neural network model forced the robot to strongly synchronize to any human tempo (within the range of possible frequencies: from 0.2 to 0.5 Hz) during the interaction. This behavior clearly simulated an agent trying to intentionally coordinate. Unintentional synchronizations in humans, on the other hand, in addition to not being stable, occurred mainly within a narrow range of frequencies around the preferred one [10]. Consequently, unintentional behaviors were not simulated by the artificial agent in this study.

The second aspect concerns the human behavior. In Condition 1, the participants “had no visual or auditory feedback” so they were moving at their own pace without any external perturbation. In Conditions 4 and 5, participants were intentionally synchronizing as we explicitly asked for. In Conditions 2 and 3, even if we asked the participant to move at their preferred rhythm regardless of the robot behavior, we could not ensure the exact nature of their intention. Some could have tried to keep their pace while others could have willingly avoided synchronizing with the robot rather than to keep moving at their preferred tempo.

### Tempo matching, tempo variability and unintentional rhythmic entrainment

In the intentional Conditions (4 and 5), not surprisingly, mean periods analysis exhibited a near-perfect tempo matching (see Fig 3) which confirms human abilities to intentionally coordinate and synchronize [6]. Periods standard deviations revealed low variations in tempo in intentional conditions (see Fig 3). Tempo variability was logically higher in Condition 5 where both agents (human and robot) were trying to mutually synchronize. The fact that human variability is higher than robot’s in Condition 5 is probably due to the fact that the participants were better in leading the intention to synchronize and have a better capacity to react to tempo variations. Consequently, they varied their behavior before the robot could. To confirm or evaluate this effect, more experiments must be conducted on intentional coordination with different values of the coupling factor used to control the robot. The robot’s capacity to rapidly synchronize (e.g., the number of cycles to synchronize) is mostly dependent on the coupling factor strength. More details about the effects and importance of the coupling factor in the used neural model can be found in [46, 56]. We can hypothesize that, depending on this coupling factor, human and robot can switch from leader to follower with an irremediable effect on their tempo variability. In addition to the coupling factor, the mechanical limits of the robot which restrict drastically motor reaction times comparing to humans might limit the robot’s variability as well. In Condition 1, humans and robot mean periods were close showing that the robot was able to catch the rhythm set by the blindfolded participants. A higher tempo variability is logically observed for the robot as it was solely trying to adapt its behavior in this condition. Moreover, we can notice that human mean period in this condition is quite similar to the one of Condition 5 (intentional bi-directional) where, as for Condition 1, the human was setting the tempo. Indeed, in condition 1, even if the robot’s behavior simulated intended synchronization, the robot’s capacities to adopt subjects’ rhythms was not as good as a human coordinating intentionally. Tempo matching was not as perfect as in conditions 4 and 5. Again, limitations came from the mechanical abilities of the robot and the range of possible reachable frequencies set by the coupling factor. In fact, some participants were moving (at some moments) at tempos too slow or too fast for the robot. Regarding unintentional Conditions (2 and 3), results on Periods and their standard deviations (Fig 3) were informative about the concepts of unidirectional and bidirectional unintentional rhythmic entrainment. Compared to Condition 1, Condition 2 exhibited a significant lower mean period (Fig 3) without depicting more variability (Fig 3). These results demonstrated that, if the participant found a certain stability while trying to keep their pace in presence of rhythmic perturbation (robot’s movement at fixed tempo) they could not avoid rhythmical entrainment and were sped up by the robot’s Tempo. Condition 3 showed interestingly (Fig 3) that human and robot mutual tempo was a compromise between participants’ preferred rhythm (Conditions 1) and robot’s fixed initial one (See Conditions 2 and 4 in Fig 3). It demonstrated a clear bi-directional rhythmical entrainment as humans were sped up by the robot and inversely the robot was slowed down by the participants. Robot’s variability was slightly higher than participants’ in this condition (Fig 3). The reason behind this fact is that the behavior simulated by the robot was an intentional synchronization. The robot was the one trying continuously to adapt its tempo while human participants were keeping, as well as they could, their own pace. In summary, the important result to highlight here regarding tempo is obviously the fact that, in both unidirectional and bi-directional conditions (Conditions 2 and 3), human unintentional rhythmical entrainment was observed during the interaction confirming our hypothesis.

### Synchronization, coordination stability: Unilateral vs bi-directional coordination

Synchronization and coordination degree were assessed by circular variance and relative phase angle distribution. The results (Figs 4 and 5) ranked the conditions from low to high mean synchronization as follows: Unintentional Uni Human (Condition 2), Unintentional Uni-Robot (Condition 1), Unintentional Bi Robot/Human (Condition 3), Intentional Uni Human (Condition 4) and finally Intended Bi Robot/Human. It is important to note the poor level of synchronization in Condition 2 (Unintentional Uni-Human). Although we cannot ensure that participants were not trying to coordinate with the robot (rather than just keeping their pace), we believe they were not intentionally synchronized (regarding the low level of synchronization). We can consequently argue that the rhythmical entrainment discussed above in Conditions 2 and 3 was unintentional. Stable synchronization was found for Condition 1 meaning that the robot was mainly able to coordinate its behavior (frequency and phase) with the participants. Circular variance (Fig 4) showed that synchronization was slightly better in Condition 3 (UnintentionalBi) compared to Condition 1 where the robot exhibited the same behavior. Relative phase Distribution (Fig 5) revealed on the other hand a significant higher stability in movement synchronization pattern in Condition 3 compared to Condition 1. It demonstrates the bi-directional entrainment contribution of the human/robot interpersonal coordination. The same phenomenon can be seen in intentional conditions with a better performance in terms of synchronization stability in Condition 5 (bi-directional) compared to Condition 4 (unidirectional). The difference is, however, less significant here compared to the unintentional conditions because of the high capacity of human subjects to intentionally synchronize in a very stable way. The main outcome to note here is that, as in human-human interpersonal coordination, better synchronization is obtained in case of mutual and bi-directional entrainment for both unintentional and intentional conditions. Besides and not surprisingly, as in human-human interpersonal coordination, human intentional coordination is far more stable than unintentional one in HRI.

## Limitations and conclusion

The current study demonstrated that the characteristics of human-human interpersonal coordination can clearly be replicated and confirmed in the case of HRI. More precisely the obtained results showed that, as for human-human interpersonal coordination, while interacting with a robot (i) human unintentional entrainment effect is present (Condition 2, the robot frequency is at a fixed value), (ii) bi-directional entrainment brings more stability and better synchronization for both unintentional and intentional coordination (Conditions 3 and 5), (ii) intentional coordination is stronger and more stable than unintentional coordination. However, other issues can be discussed based on our results. Although we have demonstrated the existence of human unintentional rhythmical entrainment and synchronization in HRI, this study does not detail the way this phenomenon occurs. Consequently, we cannot precisely compare the whole aspects of human-human interpersonal coordination with HRI. In fact, many questions can rise bringing interesting possible perspectives to this work. More attention needs to be paid to human participants preferred frequencies in future experiments. In fact, as mentioned in the Introduction, the literature depicted that the best range for observing unintentional coordination is when the two participants move within +-10% of their preferred frequency [10]. In future work, we will consequently aim to evaluate participants’ preferred frequencies and control the robot in a way that it can only coordinate in a small range of tempos around the preferred one. This behavior is quite achievable with the neural model used here by setting the robot initial frequency to the participants’ preferred one and by using a very low coupling factor limiting the robot’s rhythmical entrainment within a small range of tempos around the initial one. In this way, we would be better able to simulate robot’s unintentional coordination and better analyze, for example, bidirectional entrainment in conditions where both agents (human and robot) are acting in a similar way (unintentional behavior). Similarly, as in human-human interpersonal coordination, we could better analyze the effects of entrainment signal amplitudes. For instance, Varlet et al. (2012) showed that stimulus amplitude influences unintended entrainment [57]. More precisely, a better synchronization performance was the consequences of larger stimulus amplitudes of increased visual activity, but was also due to larger stimulus amplitudes resulting in an increase in the movement amplitude of an individual’s limb movements. In our experiments, humans are entrained visually by the robot arm movement meaning that the robot’s size or the distance between the robot and the participants can play a role on rhythmical entrainment force through optical flow amplitude variations which highly depend on the distance between the agents. Effects of phase shifting (e.g., [58] in unintentional entrainment could also be studied by making the robot matching the human tempo (frequency) but with a given forced phase shift. In human-human studies, the phase shift is often used to indicate which participant lead the synchronization (e.g., [58]. It could then be of interest in human-robot interaction to manipulate the phase shift of the robot in order to enhance (or reduce) the level of synchronization as well as the role of participant (as leader or follower). Additionally, we probably need a more “social” task to better analyze interpersonal coordination in a more natural HRI. Such a change obviously will bring a greater challenge in dealing with both the robots’ mechanical capacities as well as the efficiency of the computational model controlling it. In the future one might investigate whether the robot’s physical appearance/mechanical aspect can affect rhythmic entrainment in HRI. Some studies have suggested that humanoid robots are more likely able to have motor influence on human behavior [38]. Our study cannot respond to this question as we used only one type of humanoid robot. However, we hypothesize that physical appearance of the robot may not have a significative impact especially in the case of unintentional entrainment given that humans can be entrained by a simple moving dot [11]. On the contrary, we argue that motor and kinematic behavior of the robot is drastically important. More precisely, stable interpersonal coordination needs a persistent balance between motor and sensors signals exchanged by the interacting agents. Consequently, the computational model used for robot control must be chosen very carefully especially for studying unintentional entrainment. A first possibility is to consider the modeling of this phenomenon of rhythmic entrainment by complex predictive approaches allowing (with or without learning) the anticipation, and therefore, the correction and automatic control of the robot motion dynamics. Another possibility is inspired by theories of dynamical systems: Interpersonal coordination between two agents is explained by the presence of two dynamic systems, each with their own rhythm and influencing each other constantly as in the classic HKB model [8]. The model used here derives from dynamical system theory, both agents (human and robot) are considered as two oscillators (oscillatory motor control) exchanging continuously energy (visual motion energy) and converging to common frequency and phase if synchronization occurs. Without questioning the usefulness of predictive models in social interactions, we believe this approach to modeling rhythmical entrainment effect by dynamical systems better defines and explains the unintentional and inevitable nature of this synchronization phenomenon. The dynamical systems approach suggests that unintentional synchronization must be taken into account as an existing very low-level sensorimotor reflex rather than a result of more complex or high-level cognitive process. This assumption is supported by the phenomenon presence while the human is rhythmically entrained by a simple artificial agent (in our study) or even a moving dot [11]. The presence of such a phenomenon in human robot interaction, as proved here, must question the manner to model robots, specifically in the case of those called “social” robots which are supposed to real time interact with human partners. In fact, despite its overwhelming presence and importance in human interactions, unintentional rhythmic entrainment is not considered in robotics. Most of the existing architectures controlling robots are actually based on optimal control policies. Very few include low level and persistent sensorimotor reflexes as unintentional rhythmic entrainment of the motor controller (by sensory signals). We think that adding to the existing solutions, in a careful manner, low level sensorimotor reflexes simulating unintentional rhythmic entrainment can enhance intuitiveness and naturalness in HRI. These different questions and perspectives show indeed some limitations of the study presented here but also highlight the interest of using, in a near future, artificial agents to investigate human interpersonal coordination as well as define the characteristics needed to better model future social robots.

## Supporting information

S1 File Data set. Archive File including the SPSS files used for the analyses in the Results section.
(ZIP)
